# Prediction of HLA Class II Alleles Using SNPs in an African Population

**DOI:** 10.1371/journal.pone.0040206

**Published:** 2012-06-28

**Authors:** Fasil Tekola Ayele, Elena Hailu, Chris Finan, Abraham Aseffa, Gail Davey, Melanie J. Newport, Charles N. Rotimi, Adebowale Adeyemo

**Affiliations:** 1 Center for Research on Genomics and Global Health, National Human Genome Research Institute, National Institutes of Health, Bethesda, Maryland, United States of America; 2 Armauer Hansen Research Institute, Addis Ababa, Ethiopia; 3 Brighton and Sussex Medical School, Brighton, United Kingdom; University of Alabama at Birmingham, United States of America

## Abstract

**Background:**

Despite the importance of the human leukocyte antigen (HLA) gene locus in research and clinical practice, direct HLA typing is laborious and expensive. Furthermore, the analysis requires specialized software and expertise which are unavailable in most developing country settings. Recently, *in silico* methods have been developed for predicting HLA alleles using single nucleotide polymorphisms (SNPs). However, the utility of these methods in African populations has not been systematically evaluated.

**Methodology/Principal Findings:**

In the present study, we investigate prediction of HLA class II (HLA-DRB1 and HLA-DQB1) alleles using SNPs in the Wolaita population, southern Ethiopia. The subjects comprised 297 Ethiopians with genome-wide SNP data, of whom 188 had also been HLA typed and were used for training and testing the model. The 109 subjects with SNP data alone were used for empirical prediction using the multi-allelic gene prediction method. We evaluated accuracy of the prediction, agreement between predicted and HLA typed alleles, and discriminative ability of the prediction probability supplied by the model. We found that the model predicted intermediate (two-digit) resolution for HLA-DRB1 and HLA-DQB1 alleles at accuracy levels of 96% and 87%, respectively. All measures of performance showed high accuracy and reliability for prediction. The distribution of the majority of HLA alleles in the study was similar to that previously reported for the Oromo and Amhara ethnic groups from Ethiopia.

**Conclusions/Significance:**

We demonstrate that HLA class II alleles can be predicted from SNP genotype data with a high level of accuracy at intermediate (two-digit) resolution in an African population. This finding offers new opportunities for HLA studies of disease epidemiology and population genetics in developing countries.

## Introduction

The human leukocyte antigen (HLA) locus, located on chromosome 6p21.3, is the most polymorphic and gene-dense region of the human genome [1]. HLA genes play important roles in the immune system and have multiple alleles that show extensive variation across human populations. The HLA locus has been a focus for genomic research and clinical practice for several reasons: (i) it is associated with susceptibility to or resistance against several infectious, autoimmune and inflammatory diseases; (ii) it is very informative in studies of human genetic diversity; and, (iii) it is central to donor-recipient matching in tissue and organ transplantation [2]. Understanding the HLA system in African populations has unique advantages. Africa is the most genetically diverse geographical region in the world and consequently, it harbours diverse and novel HLA alleles such as the class II DQA1*0403N [3], DPA1*010602 [4], DPB1*9401, DPB1*9501 [5], DPA1*010303, and DPA1*0303 [6] alleles. HLA alleles are found to be associated with susceptibility and resistance to infectious diseases including HIV/AIDS, tuberculosis, and malaria that impose huge public health burdens in Africa [7], [8]. HLA studies have also yielded important insights into the role of pathogens in driving HLA polymorphism. For example, a study that analyzed 61 human populations across the world showed that populations that have a greater burden of pathogens show higher HLA diversity and that populations farther from Africa (geographic distance measured through landmasses from Ethiopia) are characterized by lower HLA diversity [9]. Despite such interest in HLA studies from diverse disciplines, direct typing of HLA genes is time consuming and expensive. Analysis of the results and assignment of HLA gene alleles requires special software and expertise [10], [11], [12], [13]. Of particular concern is the fact that most of the resources needed for direct typing of HLA alleles are inaccessible to research institutions and clinical centers in many developing countries, including those in Africa.

The use and acceptance of single nucleotide polymorphism (SNP) genotype data in the HLA region to predict HLA alleles is increasing, especially in non-African populations. This approach is less expensive than classical HLA typing, and in some instances the required SNP data may already have been generated through high-throughput genotyping done for large-scale genomic studies. Computational and statistical algorithms that have been developed to predict HLA alleles using SNPs include: (i) a ‘tag SNP’ method that predicts HLA alleles using up to three tagging SNPs that are in strong linkage disequilibrium with HLA genes [14], [15], (ii) an identity-by-decent (IBD) algorithm for predicting HLA gene alleles using SNP haplotypes and phased HLA data [16], [17], (iii) a unified framework for inferring HLA alleles using pedigree information, known HLA types of some individuals, and the relationship between SNP haplotyes and HLA alleles [18], (iv) an iterative method for HLA allele prediction using unphased SNP data and shared IBD segments between pairs of individuals [19], and (v) a multi-allelic gene prediction (*MAGPrediction*) method for predicting highly polymorphic gene alleles using unphased SNP genotype data [20], [21].

Despite promising reports of prediction performance of the above *in silico* methods, which were developed and validated using data from European populations, most have not been systematically evaluated in datasets generated from African populations. Only the tag SNP method has been used on an African population dataset (the YRI - Yoruba from Nigeria - in the HapMap dataset) to construct a high resolution HLA and SNP haplotype map. However, the approach has limitations for HLA inference because HLA genes are highly polymorphic and several tag SNPs need to be typed. Furthermore, two or three tag SNPs cannot usually provide the resolution needed to identify rare HLA alleles that have multiple haplotype backgrounds [16]. This problem becomes more marked in African datasets because of weaker linkage disequilibrium among neighboring loci [22], [23]. More importantly, for several reasons, informative tag SNPs that capture some of the HLA variation in one African population cannot be used as proxies for predicting HLA alleles in other African populations. Firstly, African populations have wide genetic and haplotype diversity [24], [25]. Secondly, of the several alleles of an HLA gene enumerated across global populations, only a sub-set is found in any given population [26]. Finally, novel alleles present in one population group may be absent in other population groups. In summary, the high polymorphism of HLA genes, genetic diversity among African populations and resource limitations in these settings present important challenges to direct HLA typing and/or the use of the same set of tag SNPs across different African populations.

Here, we describe the accuracy and reliability of HLA prediction from SNP data in an African population with the goal of evaluating this as a simple, inexpensive, and accurate method of determining HLA types in African populations in general. To achieve this goal, we predicted the HLA class II DRB1 and DQB1 alleles in the Wolaita population from southern Ethiopian population using genome-wide SNPs generated from a commercial genotyping array. We also describe distributions of HLA-DRB1 and HLA-DQB1 alleles in our sample using predicted and directly typed HLA data, and compare the allele distributions with available published data from two other Ethiopian ethnic groups, the Amhara and the Oromo.

## Results

### Prediction

Demographic characteristics of the HLA typed individuals included in the prediction (training and testing) were presented in **Table S1**. A set of 19 SNPs (within the region from 32,514,144 to 32,582,075 bp) and 10 SNPs (within the region from 32,606,390 to 32,643,859 bp) were selected by the *MAGPrediction* model for predicting HLA-DRB1 and HLA-DQB1 alleles, respectively. Of these SNPs, three were within the *HLA-DQB1* gene (**Table S2**).

### Evaluation of Accuracy of Prediction

At intermediate (two-digit) resolution, the prediction probabilities calculated by the model were high: 87.8% and 91.6% for HLA-DRB1, and 87.4% and 85.4% for HLA-DQB1 in the test and prediction sets, respectively. However, at high (four-digit) resolution, the prediction probabilities were <32% and the model did not output any alleles. Below we describe only intermediate resolution prediction results. The prediction accuracy of the model was also high: 95.5% for HLA-DRB1 and 87.0% for HLA-DQB1 (**Table 1**). The flanking boundaries determined by the objective function showed that SNPs in a region spanning ±35–40 kb of *HLA-DRB1* and *HLA-DQB1* genes were optimal for predicting the respective HLA alleles (**Fig. S1**).

**Table 1:** Prediction probability and accuracy of predicting HLA-DRB1 and -DQB1 alleles.

	Intermediate (2-digit) resolution		High (4-digit) resolution	
	Prediction probability,mean (S.D.)	accuracy	Prediction probability, mean (S.D.)	Accuracy
**HLA-DRB1**				
Test set	0.88 (0.17)	0.96	0.30 (0.17)	NA^a^
Prediction set	0.92 (0.15)	–	0.32 (0.21)	NA
**HLA-DQB1**				
Test set	0.87 (0.16	0.87	0.31 (0.11)	NA
Prediction set	0.85 (0.16)	–	0.31 (0.22)	NA

a The symbol NA means not available because prediction was not performed.

**Figure 1:**
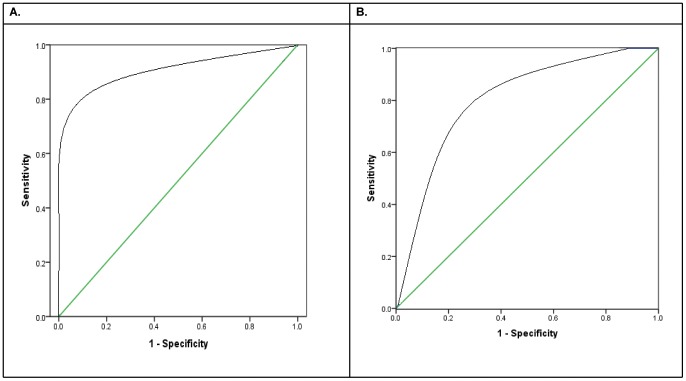
Receiver operating characteristic curves of correct HLA allele predictions at different prediction probability cut-off points. (A) HLA-DRB1, AUC = 0.86 (95% CI = 0.78–0.99), P = 0.001. (B) HLA-DQB1, AUC = 0.73 (95% CI = 0.59–0.87), P = 0.002.

The accuracy of the prediction by the intermediate resolution model was high. The sensitivity, specificity, positive predictive value (PPV), and negative predictive value (NPV) of the model was ∼100% for the majority of HLA-DRB1 alleles. The model also has high diagnostic validity for HLA-DQB1: sensitivity (77.8–100%), specificity (95.3–99.4%), PPV (77.4–92.7%), and NPV (91.7–100%) (**Table 2**). The predicted and directly typed HLA alleles had high level of agreement: κ = 0.95 (95% CI = 0.91–0.98) for HLA-DRB1 and κ = 0.83 (95% CI = 0.77–0.90) for HLA-DQB1 (**Table 3**). The receiver operating characteristic (ROC) curve showed that the model had “excellent” [27] discriminative ability for HLA-DRB1 (area under the curve [AUC] = 0.86, SE = 0.07, 95% CI = 0.78–0.99). For HLA-DQB1, the model had “good” [27] discriminative ability (AUC = 0.73, SE = 0.03, 95% CI = 0.59–0.87) (**Fig. 1**). The proportion of individuals for which both of the predicted HLA alleles (the HLA genotype) was correct was 91% and 78% and those for which at least one of the two alleles was correct was 100% and 98.9% for HLA-DRB1 and HLA-DQB1, respectively. Next, a 10-fold cross-validation done in *WEKA* [28] using two independent algorithms showed the average accuracy of prediction of HLA-DQB1 and HLA-DRB1 to be 0.81 and 0.94 and the AUC to be 0.88 and 0.93, respectively (**Table S3**).

**Table 2:** Sensitivity, specificity, positive predictive value and negative predictive value of the prediction in the test set at intermediate (two-digit) HLA allele resolution.

Allele	Observed (count)	Predicted (count)	CorrectlyPredicted (count)	Sensitivity (%)	Specificity (%)	PPV^a^ (%)		NPV^a^ (%)
**HLA-DRB1**								
07	41	41	41	100	100	100	100	
15	36	36	36	100	100	100	100	
13	22	22	22	100	100	100	100	
03	19	27	19	100	94.9	70.4	100	
04	17	17	17	100	100	100	100	
08	14	12	12	85.7	100	100	98.8	
01	11	11	11	100	100	100	100	
14	7	7	7	100	100	100	100	
11	6	0	0	0	100	0	96.6	
10	3	3	3	100	100	100	100	
Overall mean (excluding HLA-DRB1*10, HLA-DRB1*11, and HLA-DRB1*14 that had frequency <0.05 in the training set)				98.2	99.4	96.3	99.9	
**HLA-DQB1**								
02	61	55	51	83.6	96.5	92.7	91.7	
06	44	48	42	95.5	95.5	87.5	98.4	
03	28	31	24	85.7	95.3	77.4	97.2	
05	18	17	14	77.8	98.1	82.4	97.5	
04	10	11	10	100	99.4	90.9	100	
13	1	0	0	0	100	0	99.4	
Overall mean (excluding HLA-DQB1*13 that had frequency <0.05 in the training set)				88.5	97.0	86.2	97.0	

The symbols PPV and NPV mean positive predictive value and negative predictive value, respectively.

**Table 3:** Agreement between predicted and directly typed HLA alleles.

	Total allele counts	HLA alleles in agreement	Kappa, κ (95% CI)	P value
**HLA-DRB1**	176	168	0.95 (0.91,0.98)	<0.0001
**HLA-DQB1**	162	141	0.83 (0.77,0.90)	<0.0001

### HLA-DRB1 and DQB1 Allele Distributions in Three Ethiopian Ethnic Groups

A total of 16 alleles (11 DRB1 and 5 DQB1) were identified in the Wolaita ethnic group. Eleven of the 16 alleles had frequencies greater than 5%. The allele frequencies were in Hardy-Weinberg equilibrium. The three most frequently detected HLA-DRB1 alleles in the Wolaita were DRB1*15 (23.1%), DRB1*13 (19.0%), and DRB1*01 (18.8%). The three most common HLA-DQB1 alleles were DQB1*06 (35.6%), DQB1*05 (21.9%), and DQB1*02 (20.6%). The distribution of HLA alleles in the three populations is shown in **Table 4**. When considering the three Ethiopian ethnic groups (Wolaita, Oromo and Amhara), there were 15 (10 DRB1 and 5 DQB1) common HLA allelic sub-types. In addition, one Oromo individual had the DRB1*12 allele and one Wolaita individual had the DQB1*01 allele. Of the 15 HLA alleles common to the three groups, the differences in frequency of nine were not statistically significant. However, significant frequency differences were observed for the following alleles: the frequencies of DRB1*01 and DQB1*05 were higher in the Wolaita (18.8% and 21.9%) and Amhara (13.3% and 16.8%) than the Oromo ethnic group (5.4% and 8.4%); the frequency of DRB1*15 was higher and that of DRB1*07 and DQB1*02 was lower in the Wolaita (23.1%, 14%, and 20.6%) than the Oromo (12.1%, 21.7%, and 33.7%); and Amhara (10.7%, 20.4%, and 30.1%). DRB1*04 was significantly less frequent in the Wolaita (6.6%) than the Oromo (16.3%). The heterozygosity levels of DRB1 and DQB1 in Wolaita were 0.86 and 0.76, respectively, and were similar to those in the Amhara (0.86, and 0.75) and Oromo (0.84, and 0.73). In Wolaita, the inbreeding coefficient of an individual relative to the population (F_IS_ = −0.003) was not statistically significant (P = 0.65).

**Table 4:** Distribution of HLA-DRB1 and HLA-DQB1 alleles in the Wolaita, and comparison of their frequencies with the Ethiopian Oromo and Amhara ethnic groups.

	Allele count (%)^a^					P value based on Chi-square test					
	Wolaita(predicted and typed) n = 197	Oromo^b^n = 83		Amhara^b^n = 98		Wolaitavs.Oromo		Wolaita vs. Amhara		Amharavs.Oromo	
**HLA-DRB1**											
01	74 (18.8)	9 (5.4)	26 (13.3)		<0.0001			0.09		0.01	
03	29 (7.4)	18 (10.8)	16 (8.2)		0.18			0.73		0.38	
04	26 (6.6)	27 (16.3)	22 (11.2)		0.0004			0.05		0.16	
07	55 (14.0)	36 (21.7)	40 (20.4)		0.02			0.05		0.77	
08	24 (6.1)	9 (5.4)	16 (8.2)		0.76			0.35		0.31	
10	6 (1.5)	4 (2.4)	6 (3.1)		0.47			0.21		0.71	
11	6 (1.5)	4 (2.4)	5 (2.6)		0.47			0.38		0.93	
13	75 (19.0)	37 (22.3)	42 (21.4)		0.38			0.49		0.84	
14	8 (2.0)	1 (0.6)	2 (1.0)		0.22			0.37		0.66	
15	91 (23.1)	20 (12.1)	21 (10.7)		0.003			0.0003		0.69	
12	0	1 (0.6)	0		NAc			NA		NA	
**HLA-DQB1**											
02	77 (20.6)	56 (33.7)		59 (30.1)			0.001		0.01		0.46
03	69 (18.5)	35 (21.1)		40 (20.4)			0.48		0.57		0.88
04	12 (3.2)	8 (4.8)		5 (2.6)			0.36		0.66		0.25
05	82 (21.9)	14 (8.4)		33 (16.8)			0.0002		0.15		0.02
06	133 (35.6)	53 (31.9)		59 (30.1)			0.41		0.19		0.71
01	1 (0.3)	0		0			NA		NA		NA

a The denominator for each proportion is total number of chromosomes = 2 n.

b Source: HLA Nomenclature, http://www.allelefrequencies.net/.

c The symbol NA means P values were not calculated because HLA-DRB1*12 and HLA-DQB1*01 were absent in Oromo and Amhara.

## Discussion

This study demonstrated high accuracy of two-digit HLA prediction using a small set of SNPs in an Ethiopian population. This suggests that HLA prediction may provide a simpler and less expensive alternative to direct typing of HLA genes in an African population. We were able to predict intermediate (two-digit) resolution HLA-DRB1 and HLA-DQB1 alleles at accuracies of 95.5% and 87.0% using a set of 19 SNPs and 10 SNPs, respectively. Of the 29 SNPs selected in our prediction, six (i.e., rs2516049, rs477515, rs660895, rs532098, rs1063355, rs660895) belonged to the set of SNPs identified during construction or validation of different *in silico* HLA prediction methods [14], [16], [20] (**Table S4**). Compared to the prediction accuracy of the *MAGPrediction* model in the European population dataset, its accuracy in our dataset was higher for HLA-DRB1 and lower for HLA-DQB1 at confidence threshold (CT) = 0 [20]. The sensitivity, specificity, PPV and NPV of our prediction was on average 98.2%, 99.4%, 96.3%, and 99.9% for 10 HLA-DRB1 alleles and 88.5%, 97.0%, 86.2% and 97.0% for six HLA-DQB1 alleles common in our studied population. The levels of agreement (i.e., κ = 95% and κ = 83% for HLA-DRB1 and HLA-DQB1, respectively) between the predicted and experimentally determined HLA alleles were “almost perfect” according to the classification of strength of agreement by Landis and Koch [27]. The AUC of the ROC curve showed that the model had reliable discriminatory ability for both accurate and inaccurate predictions. Better accuracy and specificity could be gained by increasing the CT for the prediction probability. For example, as can be seen in **Fig. 1**, increasing CT to 0.75 in case of HLA-DRB1 and to 0.85 in case of HLA-DQB1 improves both accuracy and specificity with minimal loss in sensitivity.

While performance of the prediction at intermediate resolution was robust, there was a sharp fall in accuracy for high resolution HLA prediction. Therefore, this model is limited for clinical and/or research applications that require four-digit resolution. As has been suggested, imputation using population specific dense SNP data may improve the prediction accuracy [21]. However, we were unable to conduct imputation in the present study because of the absence of appropriate reference sequence variation data. The extensive HLA genetic diversity in African populations (for an example please see **Fig. S2**) means there is a need to study whether the SNPs selected in our samples can be transferred, and the HLA prediction method applies to other African populations.

The findings showed that the distribution of the majority of HLA alleles, and the level of heterozygosity at the HLA-DRB1 and HLA-DQB1 loci was similar among the three Ethiopian ethnic groups. The present study and a previous one [29] also showed that the frequencies of HLA-DQB1*02 (a risk allele for podoconiosis) and HLA-DRB1*13 (a protective allele against podoconiosis) in the Ethiopian population were among the highest in Sub-Saharan Africa. HLA-DQB1*02 had the third highest frequency in the Ethiopian ethnic groups following Burkina Faso’s Fulani (DQB1*0201, 36.0%) and Central African Republic’s Aka Pygmy group (DQB1*0201, 36.9%). The Fulani from Burkina Faso and The Gambia share the distribution of other specific HLA alleles closely with the Amhara and Oromo of Ethiopia [30]. Likewise, HLA-DRB1*1302, a rare allele in the majority of the world’s populations, has the second and third highest frequencies in the Ethiopian Amhara (17.3%) and Oromo (16.9%) following Saudi Arabia (19.6%). The Ethiopian ethnic groups had the highest observed frequencies of HLA-DRB1*13 compared with other populations in Sub-Saharan Africa (http://www.allelefrequencies.net/). Interestingly, HLA-DQB1*02 is implicated in several autoimmune and infectious diseases [2]. The Fulani mount a stronger humoral immune response to malaria evidenced by higher levels of antibodies against several *P.falciparum* antigens, and are less susceptible to the disease than other ethnic groups in neighboring areas [31], [32], [33]. This suggests that the high frequency of HLA-DQB1*02 observed among the Fulani may be related to the enhanced immune reactivity reported in this ethnic group [30]. Similarly the DRB1*13-DQB1*05 haplotype was associated with protection against severe malaria in the Gambian population and HLA-DRB1*13 was reported to be associated with protection against persistent hepatitis B infection [34]. It is estimated that malaria is endemic in three-quarters of the landmass of Ethiopia predisposing over two-thirds of the population to the disease [35]. The Amhara, Oromo and Wolaita ethnic groups form 64% of the total population of Ethiopia [36], and podoconiosis is common in regions predominated by these ethnic groups [37], [38], [39]. Together, these data raise the hypothesis that pathogen-driven selective forces, particularly malaria (believed to have exerted a selective pressure on the immune system [40]), induced the high frequency of the HLA-DQB1*02 and HLA-DRB1*13 alleles in the Ethiopian population.

In conclusion, we have demonstrated that HLA class II alleles can be predicted with high accuracy at intermediate (two-digit) resolution in an African population using SNP genotype data. These findings strongly suggest that the prediction model for HLA alleles described here is promising as an epidemiological tool for studying HLA associated diseases, understanding the role of pathogens in human HLA polymorphism, and population screening programs involving HLA testing in African populations.

## Materials and Methods

### Ethics Statement

Ethics approval was obtained from ethics review committees of the Medical Faculty of Addis Ababa University, AHRI/ALERT and the Ethiopian Ministry of Science and Technology. Written informed consent was obtained from all participants.

### Datasets

Data were obtained from 297 unrelated individuals enrolled into a genetic epidemiology study on podoconiosis (endemic non-filarial elephantiasis [41], [42]) from Wolaita zone, southern Ethiopia. SNP genotyping was done by deCODE Genetics using the Illumina Human610-Quad Bead Chip that contains more than 620,000 SNPs. After quality filters that removed SNPs with call rates <95%, we extracted 3,537 SNPs in the extended HLA region (chromosome 6∶28,799,220–34,204,868) [43] to generate a set of informative SNPs for HLA allele prediction. *HLA-DRB1* and *HLA-DQB1* typing data generated using the high definition Luminex® xMAP® technology, which analyses PCR-sequence specific oligonucleotide (PCR-SSO) amplified DNA samples, was also available in 188 subjects and was used to validate the HLA allele predictions [11]. Finally, we compared HLA allele frequency distributions between the population group in our dataset and unrelated and apparently healthy children of Oromo (n = 83) and Amhara (n = 98) ethnicity from central Ethiopia (http://www.allelefrequencies.net/). The mean age of the Oromo and Amhara subjects was 8.2 years, and ranges between 4 and 12 years [29], [44].

### The Multi-Allelic Gene Prediction Model

For prediction, we selected the Multi-allelic Gene Prediction model (*MAGPrediction -* http://qge.fhcrc.org/MAGprediction/) [20], [21] because it includes important factors that influence accurate HLA allele prediction including ethnicity, genotyping platforms and use of both experimentally genotyped and imputed SNPs. A more detailed description of the model can be found in Li *et al*, 2011 [20]. In brief, *MAGPrediction* is a general method for predicting highly polymorphic gene alleles using unphased SNP genotype data. Using a training dataset, the model selects informative non-redundant SNPs by employing a combined forward selection and backward elimination scheme, which starts with SNPs within an HLA gene and gradually extends to flanking regions. This procedure selects a boundary where the objective function achieves a minimum value while maintaining maximum accuracy. The SNP selection process is evaluated by an objective function (the negative log likelihood of predictive probabilities). The model calculates a range of prediction probabilities constructed based on Bayesian probability theorem, each probability corresponding to one possible pair of alleles for each individual at CTs supplied by the user. The original model was trained and validated using European population data from the Fred Hutchinson Cancer Research Center (FHCRC). The predictive model achieved intermediate and high resolution accuracies ranging, respectively, from 97–100% and 95–97% for HLA-A, from 96–98% and 94–96% for HLA-B, 98% and 97–98% for HLA-C, from 93–97% and 79–96% for HLA-DRB1, and from 97–98% and 83–95% for HLA-DQB1 [20], [21].

### Data Analysis

Based on the MAGPrediction program, we used predicted alleles obtained at the highest prediction probability, irrespective of its value, and we assumed a CT of 0, meaning all samples were predicted. Our training set included the SNP genotype and HLA alleles of 94 randomly selected individuals, of whom 87 and 77 had high resolution (i.e., amino acid level variation or four-digit resolution) HLA-DRB1 and HLA-DQB1 data, respectively. Using the training set the model selected a set of SNPs as informative markers for HLA-DRB1 and HLA-DQB1 allele prediction. Using these SNPs, we predicted HLA-DRB1 and HLA-DQB1 alleles in a test set comprising 94 individuals of whom 92 had both SNP genotypes and high-resolution HLA-DRB1 and HLA-DQB1 alleles. The intermediate-resolution (i.e., allelic group designation or two-digit resolution) HLA allele nomenclature was obtained by merging high-resolution HLA alleles with identical allele groups at the first two digits (http://hla.alleles.org/nomenclature/) [45].

Performance of the prediction model was evaluated in the test set by comparing predicted HLA alleles with experimentally determined alleles using the Luminex® xMAP® technology using multiple criteria as follows: (i) accuracy (i.e., the overall overlap between the predicted and observed HLA-DRB1 and HLA-DQB1 alleles), sensitivity (i.e., proportion of observed HLA alleles that were correctly predicted), specificity (i.e., for a specific allele, proportion of observed alleles that were different from the allele and different from that specific allele based on prediction), PPV (proportion of predicted specific HLA alleles that were actually observed), and NPV (proportion of predicted alleles negative for a specific HLA allele that were actually observed to be negative for the specific allele); (ii) a reliability analysis that tested the level of agreement between allele assignments by the model and the reference using Cohen’s kappa statistic; and (iii) evaluation of the ability of the model to be discriminative between correctly and incorrectly predicted alleles using an ROC curve. ROC curves plot the relationship between sensitivity (true positive fraction), on the y-axis, and 1-specificity (false positive fraction) on the x-axis, for different cut-off levels of test positivity, which in this case is a likelihood assigned by the model when predicting each individual’s HLA alleles to indicate the model’s estimate of accuracy. The AUC of the ROC curve was determined to provide the probability the model will assign higher prediction likelihood to HLA alleles that were correctly predicted than to those that were not. The AUC and its standard error (SE) were estimated using a nonparametric approach [46]. Further validation of the prediction was done using a 10-fold cross validation method (i.e., a model that splits the data into 10 equal sized pieces, and iteratively trains on 9 pieces and tests on the remainder and outputs the average) using two independent prediction algorithms (Random forest and J48) as implemented in *WEKA*, a machine learning algorithm for data mining tasks.

After evaluating performance of the prediction model, we applied the set of SNPs selected by the model to predict HLA alleles in an independent prediction set comprising 109 individuals with SNP data and no HLA allele data. Next, we compared HLA-DRB1 and HLA-DQB1 allele frequencies in a combined dataset (n = 197) comprising Wolaita ethnic individuals in the prediction set (n = 109) and previously HLA typed controls free of podoconiosis in the training set (n = 88) with those previously reported in apparently healthy children of Oromo (n = 83) and Amhara (n = 98) ethnicity from semi-urban parts of Asela town, Arsi, central Ethiopia (http://www.allelefrequencies.net/) [29], [44]. Individuals with podoconiosis (an HLA associated disease [42]) were excluded to minimize bias in allele frequency. Data were analyzed using *SPSS* version 19. Statistical significance was tested using the chi-square test and level of significance was set at 0.05. Expected heterozygosity was calculated using *FSTAT* version 2.9.3.2 [47].

## Supporting Information

Figure S1 **Objective function for HLA-DRB1 and DQB1 at intermediate (2-digit) resolution.** (A) HLA-DRB1. (B) HLA-DQB1
(PPT)

Figure S2 **Haplotype Display of HLA Class II Locus SNPs.** (A) CEU (Utah residents with Northern and Western European ancestry from the CEPH collection) from HapMap 3.2 database. (B) Wolaita, Ethiopia. (C) YRI (Yoruba in Ibadan, Nigeria) from HapMap 3.2 database.
(PPTX)

Table S1 **Basic Characteristics of the HLA Typed Subjects.**
(DOC)

Table S2 **SNPs Selected for Prediction of HLA-DRB1 and DQB1 Alleles.** Of the 19 SNPs selected for predicting HLA-DRB1 alleles, 13 were intragenic, 3 were within 47 kb upstream and 3 were within 4 kb downstream of the *HLA-DRB1* gene. Of the 10 SNPs selected for predicting HLA-DQB1 alleles, 3 were intragenic, 4 were within 26 kb upstream and 3 were within 8 kb downstream of the *HLA-DQB1* gene.
(DOC)

Table S3 **Ten-Fold Cross Validation of Prediction of HLA-DRB1 and DQB1 Alleles.**
(DOC)

Table S4 **Comparison of Selected Prediction SNPs in our Data with Published Datasets.**
(DOC)
