# Correction: Prediction of HLA Class II Alleles Using SNPs in an African Population

**DOI:** 10.1371/annotation/3529a6a2-4ba2-47dc-929e-399d441b0afa

**Published:** 2012-07-09

**Authors:** Fasil Tekola Ayele, Elena Hailu, Chris Finan, Abraham Aseffa, Gail Davey, Melanie J. Newport, Charles N. Rotimi, Adebowale Adeyemo

The first author's name is incorrectly referenced in the citation. The correct citation is: Tekola Ayele F. 

