# Supplementary figures and images for: Prediction of HLA Class II Alleles Using SNPs in an African Population
Source: PLoS One. 2012 Jun 28;7(6):e40206. doi: 10.1371/journal.pone.0040206 (PMC3386230; doi:10.1371/journal.pone.0040206)

## Slide 1
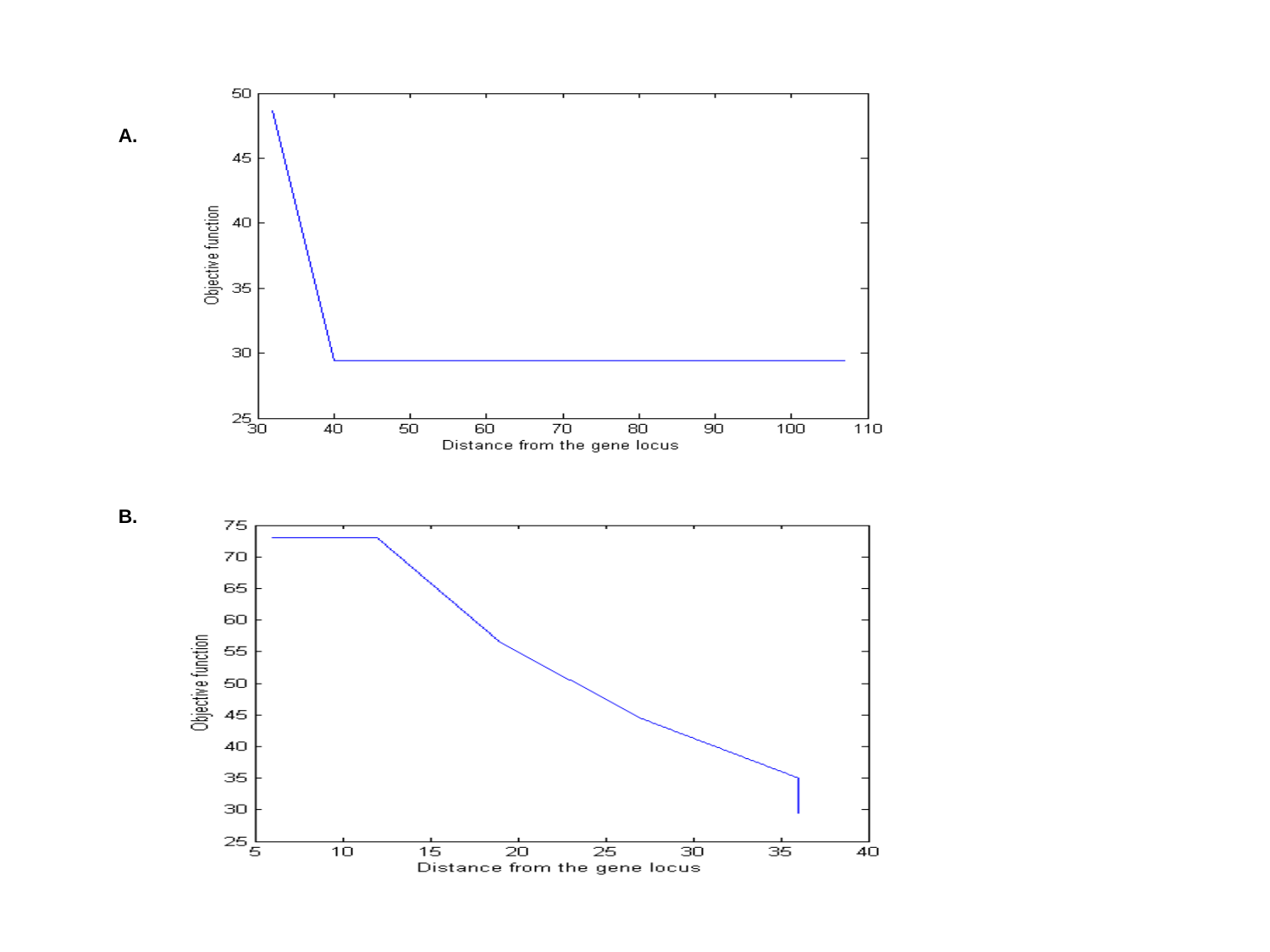

A.
B.

Supplement: Figure S1 — Objective function for HLA-DRB1 and DQB1 at intermediate (2-digit) resolution. (A) HLA-DRB1. (B) HLA-DQB1 (PPT) [file pone.0040206.s001.ppt]

## Slide 1
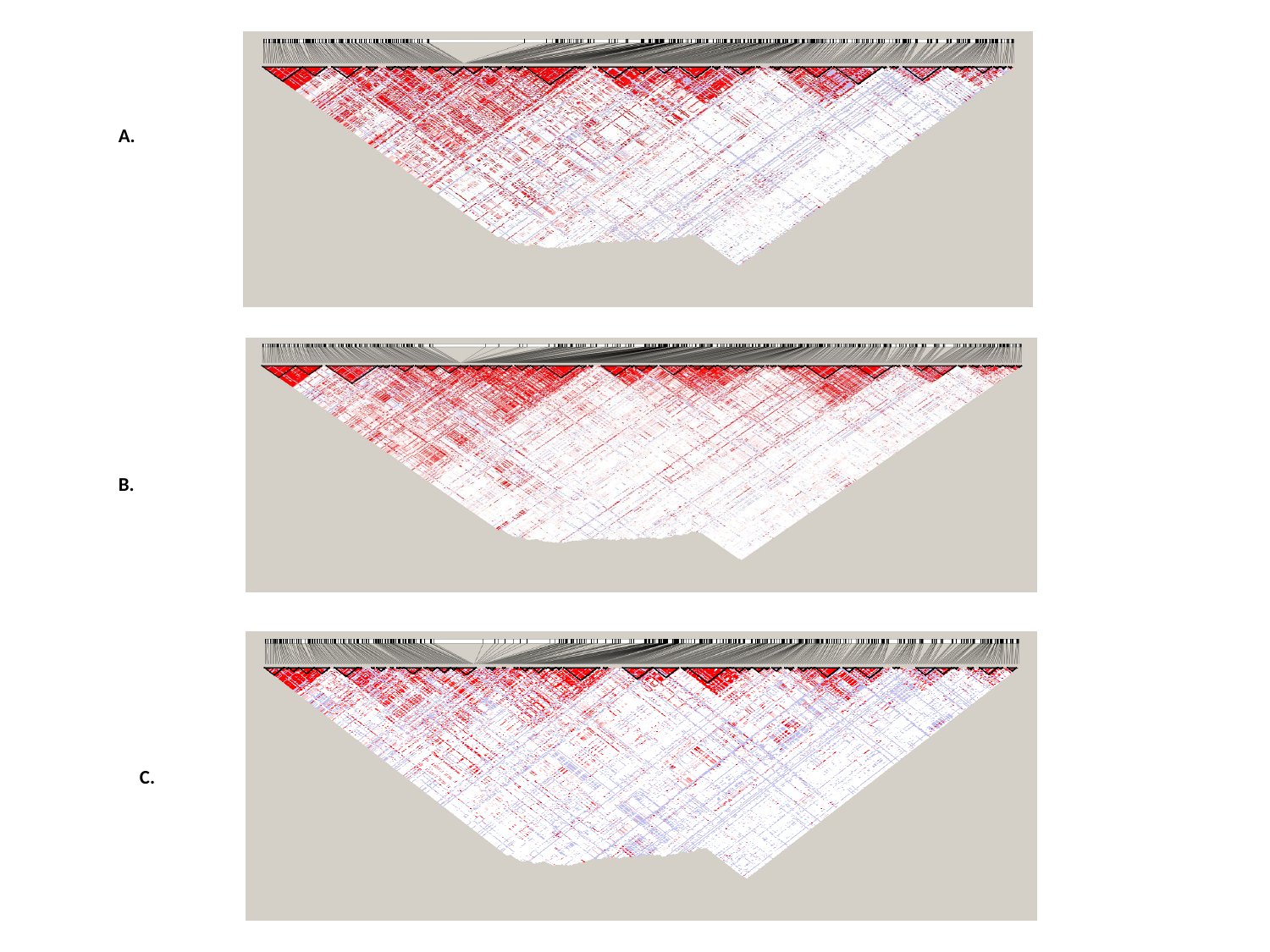

A.
B.
C.

Supplement: Figure S2 — Haplotype Display of HLA Class II Locus SNPs. (A) CEU (Utah residents with Northern and Western European ancestry from the CEPH collection) from HapMap 3.2 database. (B) Wolaita, Ethiopia. (C) YRI (Yoruba in Ibadan, Nigeria) from HapMap 3.2 database. (PPTX) [file pone.0040206.s002.pptx]
