# Supplementary material for: Prediction of HLA Class II Alleles Using SNPs in an African Population
Source: PLoS One. 2012 Jun 28;7(6):e40206. doi: 10.1371/journal.pone.0040206 (PMC3386230; doi:10.1371/journal.pone.0040206)
Supplement: Table S1 — Basic Characteristics of the HLA Typed Subjects. (DOC) [file pone.0040206.s003.doc]

| **Characteristics** | **Category (n=188)** | **Number (%)** |
| --- | --- | --- |
| Gender | Male | 90 (47.9) |
|  | Female | 98 (52.1) |
| Age in years | Mean (s.d.) | 45 (7.9) |
|  | Median (range) | 46 (18-102) |
| Occupation | Farmer | 69 (36.7) |
|  | Housewife | 52 (27.7) |
|  | Employed | 17 (9.0) |
|  | Daily labourer | 3 (1.6) |
|  | Student | 32 (17.0) |
|  | Not employed | 15 (8.0) |
| Have podoconiosis | Yes | 94 (50.0) |
|  | No | 94 (50.0) |
