# Supplementary material for: Prediction of HLA Class II Alleles Using SNPs in an African Population
Source: PLoS One. 2012 Jun 28;7(6):e40206. doi: 10.1371/journal.pone.0040206 (PMC3386230; doi:10.1371/journal.pone.0040206)
Supplement: Table S2 — SNPs Selected for Prediction of HLA-DRB1 and DQB1 Alleles. Of the 19 SNPs selected for predicting HLA-DRB1 alleles, 13 were intragenic, 3 were within 47 kb upstream and 3 were within 4 kb downstream of the HLA-DRB1 gene. Of the 10 SNPs selected for predicting HLA-DQB1 alleles, 3 were intragenic, 4 were within 26 kb upstream and 3 were within 8 kb downstream of the HLA-DQB1 gene. (DOC) [file pone.0040206.s004.doc]

| **HLA-DRB1** | | | |
| --- | --- | --- | --- |
| **No.** | **SNP** | **Position (bp)** | **Distance from**  ***HLA-DRB1* gene (bp)** |
| 1 | rs34369284 | 32514144 | -32402 |
| 2 | rs28490179 | 32519005 | -27541 |
| 3 | rs9269329 | 32540041 | -6505 |
| 4 | rs28366298 | 32560859 | 3234 |
| 5 | rs35265698 | 32561334 | 3709 |
| 6 | rs28605404 | 32569687 | 12062 |
| 7 | rs477515 | 32569691 | 12066 |
| 8 | rs2516049 | 32570400 | 12775 |
| 9 | rs9270856 | 32570839 | 13214 |
| 10 | rs2858870 | 32572251 | 14626 |
| 11 | rs9270984 | 32573991 | 16366 |
| 12 | rs9271055 | 32575369 | 17744 |
| 13 | rs9271100 | 32576478 | 18853 |
| 14 | rs660895 | 32577380 | 19755 |
| 15 | rs9271170 | 32577889 | 20264 |
| 16 | rs532098 | 32578052 | 20427 |
| 17 | rs35759989 | 32581096 | 23471 |
| 18 | rs9271300 | 32581582 | 23957 |
| 19 | rs642093 | 32582075 | 24450 |
| **HLA-DQB1** | | |  |
| **No.** | **SNP** | **Position (bp)** | **Distance from**  ***HLA-DQB1* gene (bp)** |
| 1 | rs16870207 | 32606390 | -20854 |
| 2 | rs2157337 | 32609122 | -18122 |
| 3 | rs28483633 | 32624874 | -2370 |
| 4 | rs9273349 | 32625869 | -1375 |
| 5 | rs1063355 | 32627714 | 0 |
| 6 | rs9274407 | 32632832 | 0 |
| 7 | rs2647025 | 32635949 | 0 |
| 8 | rs9274741 | 32637994 | 1834 |
| 9 | rs34115093 | 32641450 | 5290 |
| 10 | rs35465556 | 32643859 | 7699 |
