# Supplementary material for: Prediction of HLA Class II Alleles Using SNPs in an African Population
Source: PLoS One. 2012 Jun 28;7(6):e40206. doi: 10.1371/journal.pone.0040206 (PMC3386230; doi:10.1371/journal.pone.0040206)
Supplement: Table S3 — Ten-Fold Cross Validation of Prediction of HLA-DRB1 and DQB1 Alleles. (DOC) [file pone.0040206.s005.doc]

|  | **True Positive**  **Rate** | | **False Positive Rate** | | **Accuracy** | | **ROC Area** | |
| --- | --- | --- | --- | --- | --- | --- | --- | --- |
|  | **J48** | **Random forest** | **J48** | **Random forest** | **J48** | **Random forest** | **J48** | **Random forest** |
| **HLA-DQB1** |  |  |  |  |  |  |  |  |
| 02 | 0.87 | 0.85 | 0.06 | 0.05 | 0.83 | 0.85 | 0.92 | 0.92 |
| 03 | 0.80 | 0.85 | 0.08 | 0.08 | 0.82 | 0.81 | 0.92 | 0.89 |
| 04 | 0 | 0 | 0 | 0 | 0 | 0 | 0.79 | 0.75 |
| 05 | 0.85 | 0.77 | 0 | 0 | 1 | 1 | 0.9 | 0.9 |
| 06 | 1 | 1 | 0.12 | 0.19 | 0.61 | 0.84 | 0.85 | 0.89 |
| Weighted Average | 0.86 | 0.83 | 0.09 | 0.11 | 0.80 | 0.82 | 0.87 | 0.89 |
|  |  |  |  |  |  |  |  |  |
| **HLA-DRB1** |  |  |  |  |  |  |  |  |
| 01 | 1 | 1 | 0 | 0 | 1 | 1 | 0.99 | 0.99 |
| 03 | 0.78 | 0.78 | 0.03 | 0.01 | 0.81 | 0.82 | 0.86 | 0.89 |
| 04 | 0.74 | 0.82 | 0 | 0 | 1 | 1 | 0.98 | 0.99 |
| 07 | 0.83 | 0.78 | 0.007 | 0.03 | 0.95 | 0.92 | 0.92 | 0.92 |
| 08 | 0.80 | 0.79 | 0.02 | 0.01 | 0.88 | 0.95 | 0.88 | 0.85 |
| 13 | 0.82 | 0.81 | 0.03 | 0.03 | 0.92 | 0.89 | 0.87 | 0.83 |
| 15 | 1 | 1 | 0.10 | 0.14 | 0.93 | 0.91 | 0.95 | 0.98 |
| Weighted Average | 0.81 | 0.83 | 0.05 | 0.06 | 0.94 | 0.94 | 0.92 | 0.94 |
