# Supplementary material for: Prediction of HLA Class II Alleles Using SNPs in an African Population
Source: PLoS One. 2012 Jun 28;7(6):e40206. doi: 10.1371/journal.pone.0040206 (PMC3386230; doi:10.1371/journal.pone.0040206)
Supplement: Table S4 — Comparison of Selected Prediction SNPs in our Data with Published Datasets. (DOC) [file pone.0040206.s006.doc]

| **Selected Prediction SNP** | **Position** | **Prediction in our Data** | **de Bakker et al**  **(2006)^a^** | **Leslie et al**  **(2008)^b^** | **Zhang et al**  **(2011)^c^** |
| --- | --- | --- | --- | --- | --- |
| rs2516049 | 32570400 | HLA-DRB1 | HLA-DRB*1303 (YRI) | − | − |
| rs477515 | 32569691 | HLA-DRB1 | HLA-DRB*0405 (CHB)  HLA-DRB*0901 (CHB)  HLA-DRB*1303 (YRI) | − | − |
| rs660895 | 32577380 | HLA-DRB1 | HLA-DRB*0401 (CEU) | − | − |
| rs532098 | 32578052 | HLA-DRB1 | HLA-DQA*0101 (CHB) | − | − |
| rs1063355 | 32627714 | HLA-DQB1 | HLA-DQA*0103 (CHB,JPT)  HLA-DQB*0601 (JPT)  HLA-DQA*0102 (YRI,CHB) | HLA-DRB1 (YRI)  HLA-DQA1 (YRI)  HLA- DQB1 (YRI) | − |
| rs660895 | 32577380 | HLA-DRB1 | − | − | HLA-DRB1  (multiple populations) |
| The following symbols are used: CEU, Utah residents with Northern and Western European ancestry from the CEPH collection; CHB, Han Chinese in Beijing, China; JPT, Japanese in Tokyo, Japan; YRI, Yoruba in Ibadan, Nigeria  **^a^** de Bakker PI, McVean G, Sabeti PC, et al. A high-resolution HLA and SNP haplotype map for disease association studies in the extended human MHC. Nat Genet 2006; 38:1166-72.  **^b^** Leslie S, Donnelly P, McVean G. A statistical method for predicting classical HLA alleles from SNP data. Am J Hum Genet 2008; 82:48-56.  **^c^** Zhang XC, Li SS, Wang H, Hansen JA, Zhao LP. Empirical evaluations of analytical issues arising from predicting HLA alleles using multiple SNPs. BMC Genet 2011; 12:39. | | | | | |
